# Surface properties of glass micropipettes and their effect on biological studies

**DOI:** 10.1186/1556-276X-6-401

**Published:** 2011-05-31

**Authors:** Majid Malboubi, Yuchun Gu, Kyle Jiang

**Affiliations:** 1 School of Mechanical Engineering, The University of Birmingham, Edgbaston, Birmingham, B15 2TT UK; 2 IMM, Peking University, 5 Yiheyuan Road Beijing, 100871 China

## Abstract

In this paper, an investigation on surface properties of glass micropipettes and their effect on biological applications is reported. Pipettes were pulled under different pulling conditions and the effect of each pulling parameter was analyzed. SEM stereoscopic technique was used to reveal the surface roughness properties of pipette tip and pipette inner wall in 3D. More than 20 pipettes were reconstructed. Pipette heads were split open using focused ion beam (FIB) milling for access to the inner walls. It is found that surface roughness parameters are strongly related on the tip size. Bigger pipettes have higher average surface roughness and lower developed interfacial area ratio. Furthermore, the autocorrelation of roughness model of the inner surface shows that the inner surface does not have any tendency of orientation and is not affected by pulling direction. To investigate the effect of surface roughness properties on biological applications, patch-clamping tests were carried out by conventional and FIB-polished pipettes. The results of the experiments show that polished pipettes make significantly better seals. The results of this work are of important reference value for achieving pipettes with desired surface properties and can be used to explain biological phenomenon such as giga-seal formation.

## Introduction

Since Barber (1902) used a glass pipette as an intracellular microelectrode [1], micropipettes have become an essential tool for biological studies. Dozens of pipettes may be used by an individual in a single day. A micropipette works as a bridge between microscopic biological samples and macroscopic measuring devices, most often by forming a liquid channel for signal acquisition. To date, micropipettes have been used for many applications, most notably controlled delivery of liquids, genes, or sperms to the target [2-4], fertilization studies [5], intracellular measurements [1], voltage, current and patch-clamp studies [6,7]. In many of these applications, a smooth tip is preferred because it reduces the chance of tip contamination and damage to delicate biological samples [5]. Recent development in microengineering and nanosciences has found many applications of micro/nanopipettes, such as generating microdroplets [8], single-molecule fluorescence tracking [2], creating nanoscale features by nanolithography and nanowriting methods [9], and nanosensing in scanning probe microscopy [10]. Although there are many studies in the literature on the shapes and geometries of pipettes [1,6,11-15], there are no reports about numerical analysis on the effect of pulling parameters on surface roughness properties of glass micropipettes. This information is important in applications which require direct contact of pipette and samples. This paper presents an investigation on the surface roughness properties of glass micropipettes. Pipettes were pulled under different pulling conditions and the effect of each pulling parameter on surface roughness properties is investigated. SEM stereoscopic technique was used in finding the surface properties of micropipettes. More than 20 pipettes were reconstructed. To measure the inner wall surface properties of the pipettes, the pipette heads were split and cut by means of focused ion beam (FIB) milling. The results show that both of the pipette tips and pipette inner walls are rough. There is a direct correlation between tip size and surface roughness of pipette, *i.e.*, by increasing the tip size, surface roughness also increases. Autocorrelation plot of the inner wall surface of pipette shows that the surface does not have any tendency of orientation and is not affected by pulling direction. The importance of pipette surface roughness properties in biological studies is also shown. Patch-clamp technique is taken as an example of verifying the findings. Patch-clamping tests were performed using conventional and FIB-polished pipettes. It is found that polished pipettes make significantly better seals. This improvement can be explained by measured surface roughness properties and bearing area curves parameters. The results of this work are of important reference value for achieving pipettes with desired surface properties, modeling cell-pipette interactions, and explaining some biological phenomena such as giga-seal formation.

## Materials and methods

### Pulling pipettes

The puller used in the experiments was Flaming/Brown micro pipette puller (Model P-97, Sutter Instrument, Novato, CA). The six parameters on this machine for controlling the shape and size of micropipettes are heat, pull, velocity, delay, time, and pressure. Full details of these parameters can be found in manufacturer's catalog [16].

To investigate the effect of each parameter on pipette's tip surface properties, one parameter was varied whereas the others were held unchanged in every set of experiments. Delay and time are both cooling parameters. Time has quite narrow working range, whereas delay provides wider range of control. Therefore, the effect of delay is investigated. Table 1 shows values of the parameters used in the experiments. Glass micropipettes pulled from borosilicate glass tubes have an outer diameter of 1.5 mm and an inner diameter of 0.86 mm (BF150-86-10, Sutter Instrument). The filament of the puller machine was FB230B (2.0-mm-square box filament, 3.0 mm wide, Sutter Instrument). Pulling pipettes continuously will make the chamber warm and gradually decrease the heating time for subsequent pipettes. For this reason the chamber was left for 5 min to cool down after pulling every five pipettes.

**Table 1:** Pulling parameters values.

Experiment	Heat	Velocity	Pull	Delay	Pressure
Effect of heat	595, 600 605	10	0	1	500
Effect of velocity	606	4, 8, 10, 12, 14	0	1	500
Effect of pull	606	10	0, 10, 30	1	500
Effect of delay	606	10	0	1, 20, 40	500
Effect of pressure	606	10	0	1	300, 350, 400, 450

**Figure 1:**
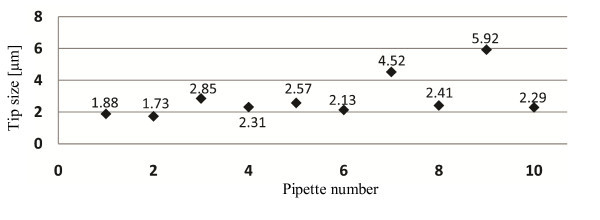
**Pipette pulling experiment records**. Ten pipettes were pulled with the same pulling parameters and their tip sizes were measured using SEM.

To test the reproducibility of the puller, ten pipettes were pulled with each set of parameters and their tip sizes were measured by SEM. Figure 1 is a summary of the statistics of the experiments. A few sudden variations in tip sizes are mainly because of the nonhomogeneities in the composition and molecular structure of borosilicate glass [1]. In the experiments, pipettes with irregular sizes, far from the expected value, were not used for reconstruction.

### 3D reconstruction of pipette tip

**Figure 2:**
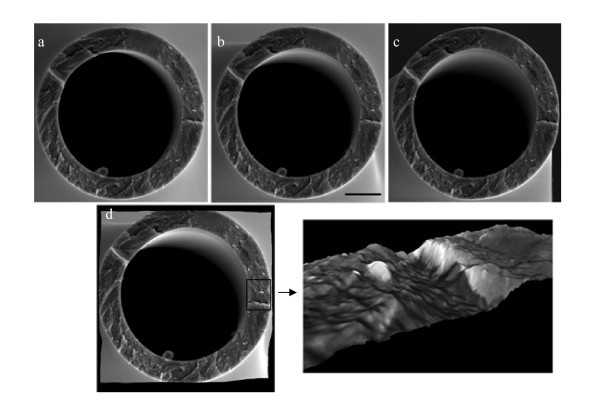
**SEM stereoscopic images captured from different angles**. -5° (**a**), 0° (**b**), and 5° (**c**), DEM created using MeX (**d**). For surface texture parameters of this pipette please see Tables 3 and 5. The bar represents 10 μm.

To determine the three-dimensional structure of pipette tips, SEM stereoscopic technique was used in the investigation [17]. To capture high-quality SEM images which satisfy stereoscopic technique requirements, glass micropipettes were coated with a thin layer of platinum (< 5 nm). The SEM machine used for 3D reconstructions was "Quanta 3D FEG" (FEI, Hillsboro, OR, USA). Three SEM images were taken from different angles by tilting the stage with respect to the electron beam direction. Differences in heights of features appear as lateral displacements in every pair of SEM images and the third dimension can be calculated from the difference between right and left images. To measure the surface characteristics, Digital Elevation Model (DEM) of the tip was created by 3D reconstruction technique using a commercial software package MeX (Alicona, Graz, Austria) [18]. Figure 2 shows the SEM stereo images used in reconstruction of a pipette and its DEM.

The important factors in SEM stereoscopic technique are magnification, tilting angle, and resolution. Since the maximum pixel resolution of the machine is limited, different magnifications, and tilting angles have been used to reconstruct every pipette's tip with maximum disparity and highest lateral and vertical resolution. Such a reconstruction could be expected to have the inaccuracy of less than 5% [19]. Table 2 gives the values of tip diameter, tilting angle, magnification, lateral resolution, and vertical resolution for three different-sized pipettes.

**Table 2:** Reconstruction information for three pipettes.

Pipette number	Tip diameter (μm)	Tilting angle (left to right)	Magnification	Lateral resolution (nm)	Vertical resolution (nm)
1	34.5	10	5,000	29	41
2	19.3	10	8,000	18.1	19.7
3	3.7	10	50,000	5.8	8.2

### Cell culture and patch-clamping experiments

Human umbilical vein endothelial cells (HUVECs) were used for patch-clamping experiments. HUVECs were cultured in Endothelial Basal Medium (EBM, CC-3121, Lonza, Basel, Switzerland) on coverslips for 2 to 3 days before the experiments. At the time of experiments, the confluence of the cells was over 80%. Incubation was done at 37°C. Patch-clamp experiments were done using axon multiclamp 700B microelectrode amplifier (Axon Instruments, Union City, CA, USA). The average opening of the pipette tips was about 1.4 μm in diameter. The backfilling solution was composed of 40 mM KCl, 96 mM K-gluconate, 4 mM K_2_ATP, 2 mM GTP, 10 mM HEPE, and at 7.2 in pH, and the bath solution was composed of 110 mM NaCl, 5 mM KCl, 1 mM MgCl_2_, 1 mM CaCl_2_, 5 mM HEPEs, 5 mM HEPE-Na, and at 7.2 in pH.

## Results and discussion

**Figure 3:**
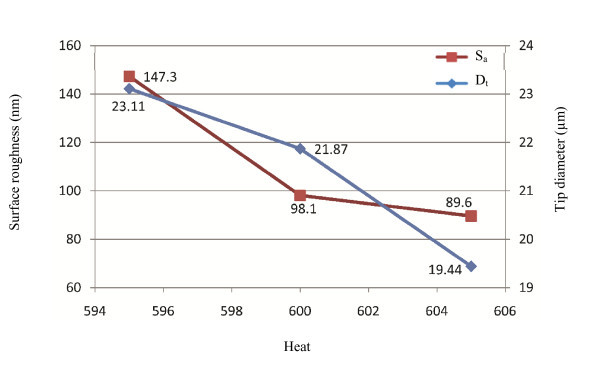
**The effect of heat on tip diameter and average surface roughness**. The heat is controlled by the level of electrical current supplied to the filament. The unit of heat is milliamp. Useful changes in heat are 5 units or more to see an effect. By increasing the heat, both of the *S*_a _and *D*_t _decreases.

**Figure 4:**
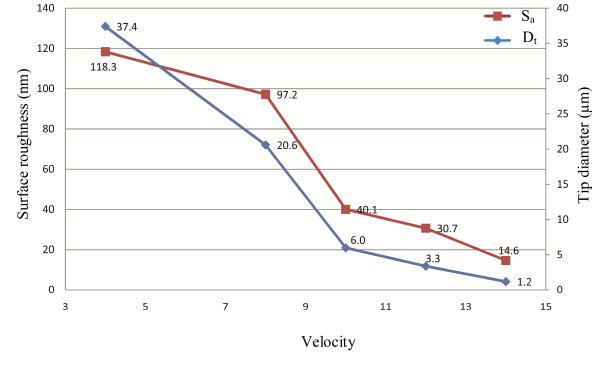
**The effect of velocity on tip diameter and average surface roughness**. This control measures the velocity of the glass carriage system as the glass softens. By increasing the velocity, both the tip size and the surface roughness decrease. The velocity has the most significant effect on the tip size and the surface roughness. A small change in velocity value decreases *S*_a _and *D*_t _rapidly.

**Figure 5:**
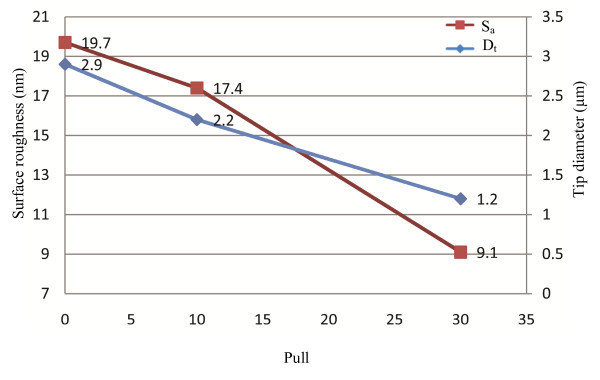
**The effect of the pull on tip diameter and average surface roughness**. This parameter controls the force of the hard pull. The amount of the pull determines the current to the pull solenoid. Useful changes in pull strength are 10 units or more to see an effect. By increasing the pull, both of the *S*_a _and *D*_t _decreases.

**Figure 6:**
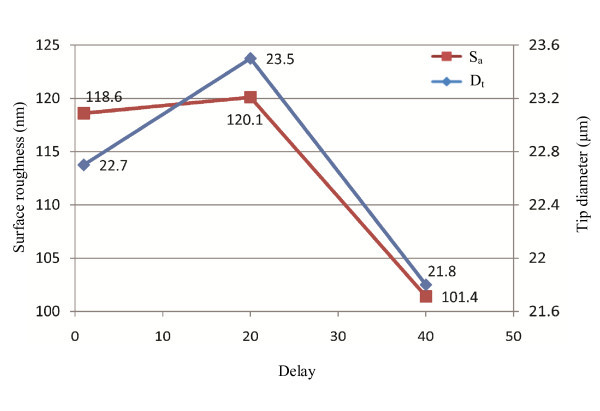
**The effect of delay on tip diameter and average surface roughness**. Delay is a cooling mode which controls the delay time between the time when the heat turns off and the time when the hard pull is activated. One unit of delay represents 1/2 ms. Delay is an effective means of controlling tip length which does not change the size of pipette tip notably.

**Figure 7:**
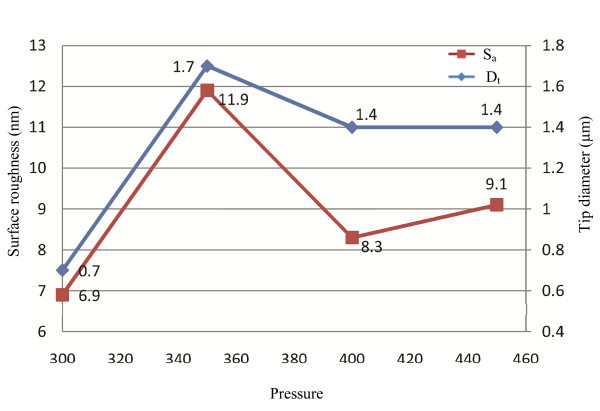
**The effect of pressure on tip diameter and average surface roughness**. This control sets the pressure generated by the air compressor during the active cooling phase of the pull cycle. The unit of pressure is psi. Changes of less than 10 units will not be noticeable. Pressure is another way of controlling tip length and does not change the size of pipette tip significantly.

Over 20 pipettes have been reconstructed in the study. The effect of each parameter is studied by investigating at least three reconstructions. Tip diameter (*D*_t_) and average surface roughness (*S*_a_) of all pipettes have been measured. Figures 3, 4, 5, 6 and 7 show correlations between pulling parameters and *D*_t _and *S*_a_.

**Figure 8:**
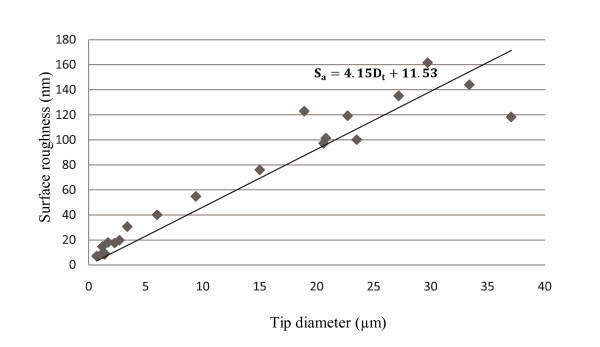
**Average surface roughness of pipette tip (*S*_a_) *versus *tip diameter (*D*_t_)**. *S*_a _is strongly dependent on *D*_t _and has a direct correlation with it. A first degree polynomial equation fitted to data suggests that *S*_a _can be estimated knowing the tip diameter of a given pipette with good approximation.

As it can be seen from Figures 3, 4, 5, 6 and 7, velocity has the most significant effect. A small increase in velocity significantly decreases *D*_t _and *S*_a_. The effects of pull and heat are very similar and not as significant as the effect of velocity. Delay and pressure are factors to change the taper length of the pipettes while keeping the tip size unchanged [16]. Increasing delay and pressure will result in a shorter taper. Although these two factors do not change tip diameter significantly, it can be seen from Figures 6 and 7 that the bigger pipette has a higher surface roughness. From Figures 3, 4, 5, 6 and 7 it can be understood that *D*_t _and *S*_a _have direct correlation. Figure 8 is obtained by plotting *D*_t _*versus S*_a _for 21 pipettes pulled with different pulling parameters. It can be seen that average surface roughness of pipette is strongly related to tip size. *D*_t _and *S*_a _have direct correlation, *i.e.*, by increasing the tip size, surface roughness also increases.

As an example, the surface properties of two pipettes with considerably different tip sizes are shown in Table 3. The bigger pipette has higher average surface roughness (*S*_a_) and lower developed interfacial ratio (*S*_dr_). The importance of these parameters in giga-seal formation will be discussed later.

**Table 3:** Tip surface properties for two pipettes having different sizes.

Name	Value (*D*_t _= 34.5 μm)	Value (*D*_t _= 3.9 μm)	Description
*S*_a_	149.1 nm	30.8 nm	Average height of selected area
*S*_q_	209.9 nm	42.0 nm	Root-mean-square height of selected area
*S*_p_	1437.4 nm	304.1 nm	Maximum peak height of selected area
*S*_v_	1,409.3 nm	238.0 nm	Maximum valley depth of selected area
*S*_z_	2,846.7 nm	542.1 nm	Maximum height of selected area
*S*_10z_	2,304.3 nm	414.59 nm	Ten-point height of selected area
*S*_sk_	-0.2118	-0.514	Skewness of selected area
*S*_ku_	7.0253	6.4208	Kurtosis of selected area
*S*_dq_	0.5767	1.3246	Root-mean-square gradient
*S*_dr_	13.129%	79.532%	Developed interfacial area ratio

**Figure 9:**
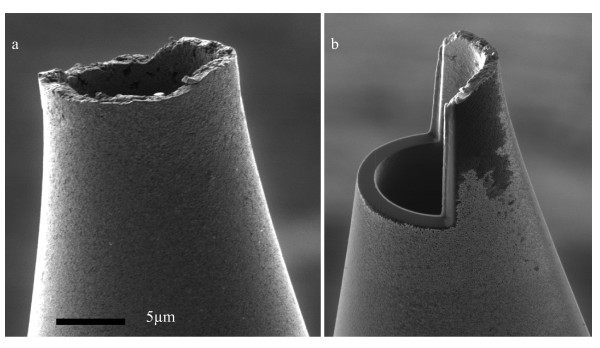
**SEM images of pipette before (a) and after (b) FIB milling**. Notice that milled surface is much smoother than the original unmodified surface.

**Figure 10:**
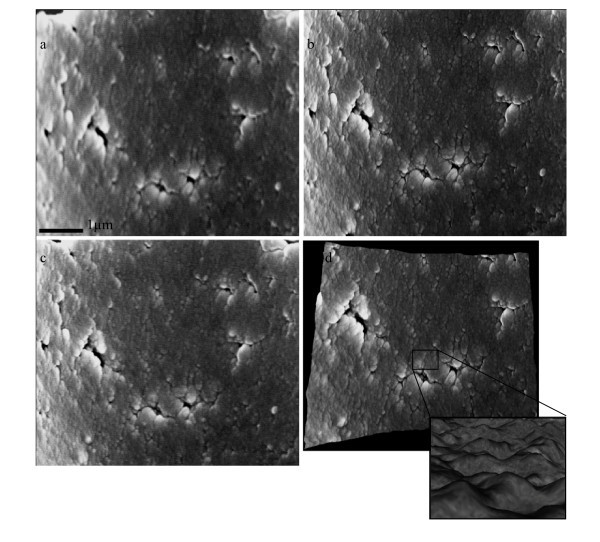
**SEM stereoscopic images of the inner wall of a pipette taken with different prospective**. (**a**) With a tilting angle of -5°, (**b**) without a tilting angle, and (**c**) with a tilting angle of 5° and (d) DEM of the pipette inner wall surface.

**Figure 11:**
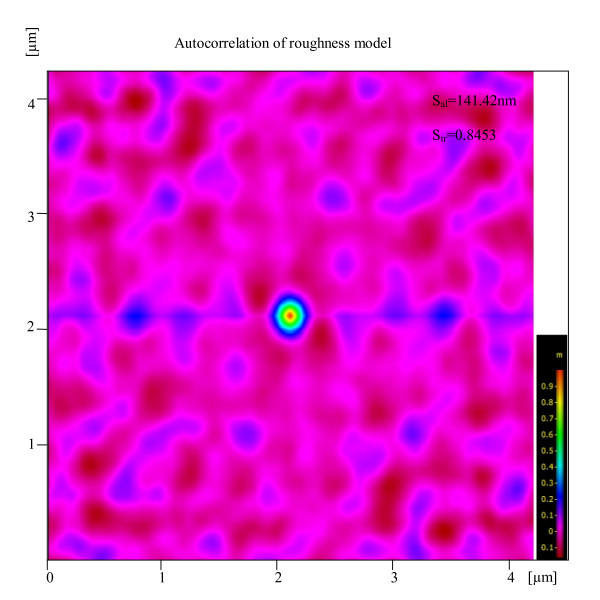
**Autocorrelation of roughness model of the surface shown in Figure 10**. The plot shows that the surface doesn't have any texture orientation. Large value of Texture Aspect Ratio of the Surface (S_tr_) indicates uniform texture in all directions *i.e.*, no defined lay. Small value of Autocorrelation Length (*S*_al_) denotes that the surface is dominated by high frequency components (see the inset in Figure 10d).

Further investigation was conducted for measuring the inner wall surface roughness properties of glass micropipettes. Two pipettes with different sizes (*D*_t _= 13 and 9 μm) were chosen. The pipettes were split and cut open using focused ion beam milling for access to the inner walls. The imaging direction was perpendicular to the cutting plane, avoiding redeposition of sputtered materials from the FIB cutting to the area. After cutting, the pipettes were turned 90° by means of a holder which was previously fabricated. Three SEM images were taken from the inside wall and 3D structures of the inner wall were obtained using MeX software. Figures 9 and 10 show an FIB-milled pipette and stereo images. Table 4 shows inner surface properties of small and big pipettes. The small pipette has a lower *S*_a _and higher *S*_dr_. To determine the effect of pulling direction on surface texture of pipette inner wall, autocorrelation of roughness model for the pipette inner surface is obtained. The autocorrelation plot, Figure 11, suggests that the surface does not have any tendency of orientation and is not affected by pulling direction.

**Table 4:** Inner wall surface properties of two different sized pipettes.

Name	Value (*D*_t _= 13 μm)	Value (*D*_t _= 9 μm)	Description
*S*_a_	31.1 nm	22.2 nm	Average height of selected area
*S*_q_	39.0 nm	28.4 nm	Root-mean-square height of selected area
*S*_p_	158.5 nm	151 nm	Maximum peak height of selected area
*S*_v_	150.3 nm	132.6 nm	Maximum valley depth of selected area
*S*_z_	308.9 nm	303.6 nm	Maximum height of selected area
*S*_10z_	273.3 nm	247.5 nm	Ten-point height of selected area
*S*_sk_	0.0267	0.186	Skewness of selected area
*S*_ku_	2.945	3.740	Kurtosis of selected area
*S*_dq_	0.616	0.838	Root-mean-square gradient
*S*_dr_	17.773%	31.965%	Developed interfacial area ratio

**Figure 12:**
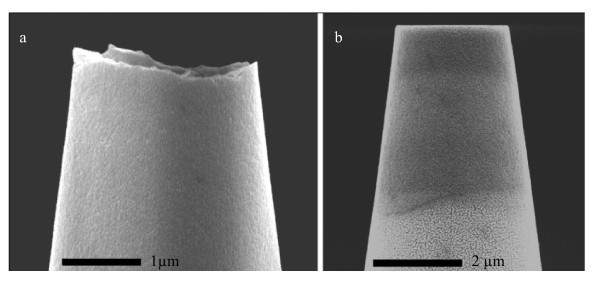
**Glass micropipette before (a) and after (b) FIB milling**.

**Figure 13:**
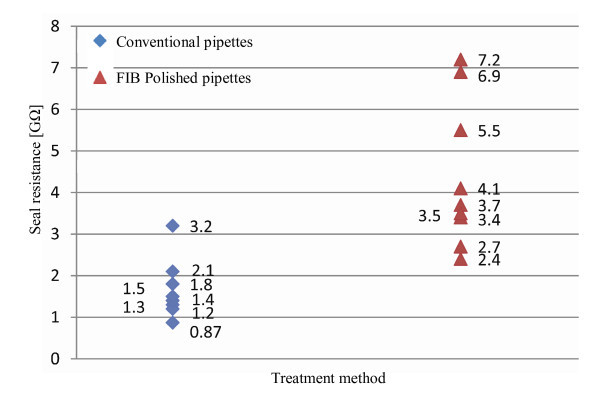
**Seal resistances for the two types of pipettes**. The results are from 20 experiments. As it can be seen, the statistics of the FIB-polished pipettes are significantly better than conventional pipettes.

**Figure 14:**
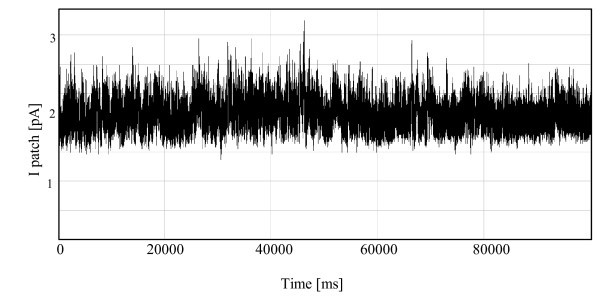
**Single-channel currents recorded from HUVECs for conventional pipettes**.

**Figure 15:**
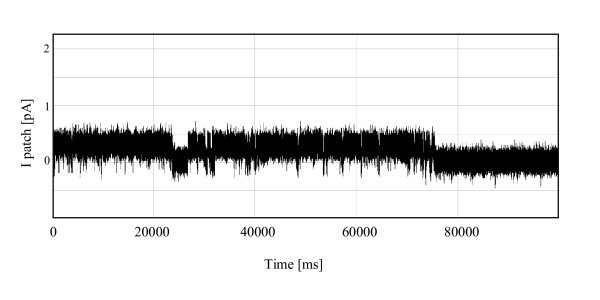
**Single-channel currents recorded from HUVECs for FIB-polished pipettes**. Comparing with Figure 14, FIB-polished pipettes resulted in a lower leakage current and noise due to the better sealing conditions of FIB-polished pipettes.

The fact that both the pipette tips and pipette inner walls are rough may help better understanding of the mechanism of applications in which pipettes are in contact with vulnerable biological samples. Patch-clamp technique is taken as an example of pipette applications. Patch-clamping suffers from current leakage between cell membrane and glass surface. Cell membrane is sucked from 5 to 100 μm into a pipette in patch clamping. Optical and electron microscope images of patches show that membrane and pipette are in close contact, but they do not show the surface topography involved in seal formation [20,21]. Surface roughness of glass micropipettes is reported in the literature to play an important role in giga-seal formation [11-13,22,23]. Normally, pipettes are fire polished before experiments to make rough tips smoother. In this study, FIB milling is used for polishing pipette. FIB polishing was found to be a more controllable process. Fire polishing requires a very good timing and positioning. Pipette end can be easily overheated which results in a closed tip or irregular tip shape. Fire polishing melts the glass and makes pipette tips smoother but it also has a blunting effect on tips which change the pipette shape and sharpness. FIB polishing allows working on the very end of pipettes (the last one micron from the tip) without changing other properties of pipettes such as shape and sharpness. To observe the importance of surface roughness of pipette on giga-seal formation, patch-clamp experiments were carried out using conventional and FIB-polished pipettes. FIB milling of pipettes leaves an ultimately smooth surface free from peaks and valleys or sharp spikes. Figure 12 shows images of a pipette before and after FIB milling. Because of the conic shape of pipette only the very end of pipette was cut during milling process in order not to change the pipette opening significantly. Ten recordings were obtained for each type of pipettes. Seal resistances are shown in Figure 13. FIB-polished pipettes formed significantly better seals which made it possible to measure single ion channel currents with considerably lower noise (see Figures 14 and 15). Higher seal resistance for polished pipettes could be explained by their better sealing potential. Contact area between pipette tip and cell membrane is higher for polished pipettes and since there are no peaks or spikes, membrane can get closer to the tip. As a result, it is more difficult for ions to escape form glass-membrane distance and higher seal are achievable.

It is well known that pipettes with a smaller opening form a better seal and lower leakage current. This can also be explained by comparing the roughness parameters of pipettes with different size openings. For instance, Table 5 shows the bearing area curve parameters of the two pipettes discussed in Table 3. Surface bearing area curve provides useful information about the peak, core and valley volumes, and fluid retention ability of the surface [24].

**Table 5:** Values of the bearing area curve for two pipette tips having different sizes.

Name	Value (*D*_t _= 34.5 μm)	Value (*D*_t _= 3.9 μm)	Description
*S*_k_	412.7 nm	85.5 nm	Core roughness depth, Height of the core material
*S*_pk_	266.3 nm	42.6 nm	Reduced peak height, mean height of the peaks above the core material
*S*_vk_	320.1 nm	66.3 nm	Reduced valley height, mean depth of the valleys below the core material
*S*_mr1_	10.91%	8.16%	Peak material component, the fraction of the surface which consists of peaks above the core material
*S*_mr2_	89.08%	84.98%	Peak material component, the fraction of the surface which will carry the load
*V*_mp_	0.013 ml/m^2^	0.002 ml/m^2^	Peak material volume of the topographic surface (ml/m^2^)
*V*_mc_	0.146 ml/m^2^	0.033 ml/m^2^	Core material volume of the topographic surface (ml/m^2^)
*V*_vc_	0.204 ml/m^2^	0.039 ml/m^2^	Core void volume of the surface (ml/m^2^)
*V*_vv_	0.028 ml/m^2^	0.006 ml/m^2^	Valley void volume of the surface (ml/m^2^)
*V*_vc_/*V*_mc_	1.410	1.174	Ratio of *V*_vc _parameter to *V*_mc _parameter

Tables 3 and 4 show that bigger pipettes have higher *S*_a _both at the tip and at the inner surface. Maximum peak to valley distance is also higher for bigger pipettes. Table 5 shows that valley void volume (*V*_vv_) is considerably high for bigger pipettes. This indicates that the bigger pipettes have more fluid retention ability. The ratio of *V*_vc_/*V*_mc _is also larger for bigger pipettes, which means that there are more voids present compared to smaller pipettes. During patch-clamp experiments, valleys and voids are filled with conductive media facilitating ion escape, increasing the leakage current and compromising the seal. By comparing the values in Tables 3 and 4, one can also find that developed interfacial area ratio (*S*_dr_) changes significantly for small and big pipettes. *S*_dr _is expressed as the percentage of additional surface area contributed by the texture as compared to an ideal plane [24]. This parameter is useful in applications which involve surface coatings and adhesion. A recent study shows that having higher *S*_dr _can promote cell adhesion significantly [25]. In giga-seal formation membrane proteins are denatured against the glass and pull the membrane closer to glass, causing a tight seal [26]. The fact that smaller pipettes have notably higher *S*_dr _at the tip and at the pipette inner wall surface means that a higher percentage of the pipette surface contributes in glass-membrane interactions. This increases the number of membrane proteins sticking to the pipette inner wall and improves the seal.

## Conclusions

In this paper, the effect of pulling parameters on the surface properties of glass micropipettes is reported. Although different pullers are being used in laboratories, they utilize the same principles; therefore the results of this study can be applied to almost all of the puller machines. More than 20 pipettes were reconstructed with SEM stereoscopic technique. The results show that surface roughness parameters of glass micropipettes are strongly related to tip size. A further inspection on the inner wall surface properties of big and small pipettes found that the bigger pipettes had higher *S*_a _and lower *S*_dr_. Autocorrelation of roughness model shows that the inner surface does not have any orientation tendency and is not affected by pulling direction. Surface roughness parameters of pipette tips have significant influence on many applications, especially when pipettes are used in contact with vulnerable biological samples, for example, in patch-clamping experiments. Single-channel currents recorded from HUVECs cells show significantly lower noise and leakage current for pipettes polished by FIB milling. This enhancement accounts for better contact conditions of polished pipettes and the fact that polished pipettes do not have valleys and voids which facilitate current leakage in the patch-clamping. The results of this study can be used to explain some observations in laboratory practice. For example, smaller pipettes make better seals because smaller pipettes have lower *S*_a _and higher *S*_dr _than bigger pipettes. The results of this work have important reference value for achieving pipettes with desired surface properties, and may also change the way of modeling cell-pipette interactions.

## Competing interests

The authors declare that they have no competing interests.

## Authors' contributions

MM conceived and designed the study, carried out the experiments, analyzed the results and drafted the manuscript. YG assisted in patch clamping experiments. KJ supervised the research, contributed in interpretation of data and revision of the manuscript. All the authors have given final approval of the version to be published.
